# Distribution of HbS Allele and Haplotypes in a Multi-Ethnic Population of Guinea Bissau, West Africa: Implications for Public Health Screening

**DOI:** 10.3389/fped.2022.826262

**Published:** 2022-04-07

**Authors:** Maddalena Martella, Mimma Campeggio, Gift Pulè, Ambroise Wonkam, Federica Menzato, Vania Munaretto, Giampietro Viola, Sabado P. Da Costa, Giulia Reggiani, Antonia Araujo, Dionisio Cumbà, Giuseppe Liotta, Laura Sainati, Fabio Riccardi, Raffaella Colombatti

**Affiliations:** ^1^Clinic of Pediatric Hematology Oncology, Department of Woman's and Child's Health, Azienda Ospedale-Università di Padova, Padova, Italy; ^2^Division of Human Genetics, University of Cape Town, Cape Town, South Africa; ^3^Hospital Raoul Follereau, Bissau, Guinea-Bissau; ^4^Clinica Pediatrica Bor, Bissau, Guinea-Bissau; ^5^Università di Tor Vergata, Rome, Italy; ^6^Aid, Health and Development Onlus, Rome, Italy

**Keywords:** HbS, sickle cell, screening, Guinea Bissau, multi-ethnic, haplotypes

## Abstract

**Background:**

Sickle Cell Disease (SCD) is an inherited condition that is widespread globally and especially in malaria-endemic West African countries. Limited epidemiological data on SCD are available for Guinea Bissau, where newborn screening is not yet implemented, routine diagnosis is not available, and care is case directed.

**Methods:**

Dried blood spots were collected from children accessing two hospitals managed by Italian Non-Governmental Organizations in the capital city of Bissau and sent to Padova for Hemoglobin (Hb) quantification through HPLC and molecular analysis. Beta globin gene analysis was performed in all; and Hb haplotype of the HbSS and HbSA patients was performed in South Africa. One hundred samples belonging to the most frequent ethnic groups were randomly selected for detection of G6PD mutations.

**Results:**

Samples from 848 consecutive children (498 males and 350 females, mean age 6.8 years) accessing the two hospitals were analyzed: 6.95% AS (4.42% allelic frequency), 0.94% SS, and 0.23% AC. 376G G6PD allelic frequency was 24%; 14.8% in AS individuals. The Senegal haplotype was the most prevalent (31%), and the proposition of chromosomes with the atypical haplotype was surprisingly high (56%).

**Conclusion:**

Our study demonstrates a significant frequency of the HbS allele in the population of Guinea Bissau supporting the implementation of screening strategies. The differences among ethnic groups can help guide targeted interventions for SCD awareness campaigns and determine priority areas for public health interventions. The pilot analysis on haplotypes reveals a large proportion of the atypical haplotype, which may be indicative of a genetically heterogeneous population.

## Introduction

Sickle Cell Disease (SCD) is an inherited condition frequently found in people of African descent. SCD is the most common monogenic disease worldwide (1) and is recognized by the World Health Organization (WHO) and the United Nations (UN) as a global public health problem. A call to action was launched by the two organizations in 2006 and 2008 due to the burden of SCD on childhood mortality in sub-Saharan Africa, as it contributes to 9-16% of deaths in children under 5 years of age on that continent (2–4). Neonatal screening, penicillin prophylaxis, immunization, and stroke prevention have dramatically increased survival for patients with SCD in developed countries but they are seldom available in Africa (5, 6), where the majority of patients live. Recent population movements have increased the number of people carrying the HbS allele coming to Europe, thereby challenging health systems to adapt to this particular group of patients with SCD (7–10).

Guinea Bissau (Supplementary Figure 1), a former Portuguese colony located in the West African Atlantic Coast, embedded between Senegal and Guinea Conakry, lies in the malaria belt. Malaria is endemic in the country with a peak transmission during the rainy season (July–October). Guinea Bissau has a population of 1,726,170 inhabitants and children aged 0–14 years constitute 39.53% (male 340,575/female 341,747). The capital of Bissau has 492,000 inhabitants (2015). The population is multi-ethnic, because more than 20 ethnic groups are present in the country, the most frequent being Balanta and Fula (11). The official national language is Portuguese-creole, although many people speak only the local languages.

In spite of the high prevalence of SCD in the surrounding West African countries, like Senegal, Mali, Guinea Conakry, and Liberia (12–14), there are limited data on the frequency of HbS (15) in Guinea Bissau. The actual health burden of SCD in the country is not known and there are no national SCD screening programs yet.

Accurate epidemiologic information is necessary for the development of sustainable health interventions and targeted health education and prevention programs in the field of SCD. The two main objectives of our pilot study were, therefore, to determine the feasibility of a SCD screening program in two hospitals in the capital city of Bissau (in terms of capacity of correct sample collection, data recording and affected patient tracking and counseling), and to determine the prevalence of SCD among the various ethnic groups of Bissau. These data, collected through a public-private partnership with non-Governmental organizations (NGOs), could help develop further research in the field of SCD in Guinea-Bissau.

## Materials and Methods

### Setting and Patient Population

This cross-sectional study took place in two hospitals in the capital city, Bissau: the Raoul Follereau Hospital (HRF, public hospital) and the Bor Children's Hospital (BCH, private hospital), both managed within the National Health System of Guinea Bissau by Italian NGOs that had already conducted projects regarding children's health in the country (16–20).

Doctors and laboratory technicians received training on SCD and sample collection in December 2012. Sample collection began during the same month, using leftover blood from samples of children who consecutively accessed both hospitals in the following 8 months. During a routine clinical visit for any health reason in which a blood sample collection was planned for other purposes, parents were offered the possibility to perform the HbS analysis utilizing the leftover blood of their child. In case of acceptance, dried blood spots were prepared from the already drawn blood samples. Basic demographics (age, gender, ethnic group, residence) were collected for each patient in a dedicated paper form and transferred to excel for data analysis.

The study was approved by the local hospital ethics board and oral informed consent was collected from the parents.

### Dried Blood Spot Collection

After oral informed consent from parents, peripheral blood samples were collected on filter paper (Whatman™ 3MM Chr Chromatography Paper). The blood spots were air dried at room temperature, individually wrapped in aluminum film, placed in a single envelope, kept in the dark, and in a location without humidity to ensure the integrity of the sample during the processes of collection, storage, and transport. Every “*collection card*” had 4–5 blood spots with demographic and ethno-linguistic records. The samples were shipped via courier to the Laboratory of Pediatric Onco-Haematology of University of Padova, Italy for processing. At arrival in Padua, all samples underwent Bglobin gene molecular analysis and High-performance Liquid Chromatography (HPLC) analysis. After genetic analysis, the DNA from HbAS and HbSS subjects was shipped to South Africa to determine the Hb haplotype.

### High Performance Liquid Chromatography HPLC Analysis

Analyses were performed on all samples with the Alliance e2695 liquid chromatography (Waters) connected with a UV-Vis detector (Waters 2489), and settled at 415 nm. The system was equipped with a 35 length × 4.6 I.D. mm, chromatography column packed with a strong cation-exchanger, 6.5 μm microparticulate (IEC SP-420N, Shodex).

The gradient was made up of mobile phase A [MES, 2-(N-morpholino) ethanesulfonic acid 20 mM, pH = 5.6] and mobile phase B (MES+ Na2SO4 0.5 M, pH = 5.6) with a flow rate of 1 ml/min. After injection of the sample, the proportion of B was increased linearly to B:A (90:10) at 5 min and to 100:0 at 9 min; finally, the mobile phase was returned to A:B 95:5 at 11 min, for equilibration.

### HbS Molecular Analysis

Genomic DNA of high molecular weight, extracted according to the QIAGEN protocol by dried blood spot, was subjected to amplification (Applied Biosiystems Thermal Cycler 2720) of the exons of the β-globin and direct sequencing. DNA samples that were heterozygous HbS/HbA were also analyzed at exon 2 and 3.

The entire coding sequence of the βglobin gene, 11p15.5 (NM_000518; OMIM#603903), was PCR-amplified with 30 cycles reactions at 94°C for 1 min, at 56°C for 1 min and at 72°C for 1 min. The reaction was done in a final volume of 50 μl comprising 50 ng DNA, Buffer II 10X, MgCl_2_ Solution 25 mM, dNTP's 10 mM, Primers 100 μM, AmpliTaq Gold DNA Polymerase 5 u/μl. The purified PCR products were subjected to direct sequencing (3500Dx Genetic Analyzer, Applied Biosystems).

### Hemoglobin Haplotype

The concentration and purity of each DNA sample was determined by Nanodrop spectrophotometry and dilutions of 100 ng/μL concentration were used for all reactions. A previously optimized 5 restriction-site PCR protocol was used for the SCD haplotype analysis. The reaction comprised 5 μL GoTaq 5X colorless buffer (Promega, USA), 1 μL dNTPs (Fermentas, USA), 0.5 μL of each primer (iDT, RSA), 0.1 μL GoTaq (Promega, USA), 1 μL DNA and made up to 25 μL with distilled water. Briefly, the reaction was cycled on the BIO-RAD Thermal cycler (BIO-RAD T100, USA) at 95°C for 5 min, 35 cycles of 94°C for 30 s, 53 or 55°C for 40 s, 72°C for 2 min and lastly kept at 72°C for 7 min. To confirm correct amplification, a 2% agarose gel (Seakem Lonza, RSA) was prepared in 1 X TBE buffer to visualize the amplicons using SYBR Safe DNA gel stain (Invitrogen, Life Technologies SA). The electrophoresis was carried out for approximately 60 min at 160 V and gel images were acquired using a protected imaging capture system (UVIPro Gold transilluminator, UK). To genotype the samples, restriction digests were done using 0.5 or 1 μL of the specific restriction enzyme (Promega, USA), 2 μL of the associated buffer (Promega, USA), 10 μL PCR product and made up to 15 μL using sterile deionized water. The digests were incubated overnight at 37°C on a thermal cycler (BIO-RAD T100, USA) and electrophoresed on a 2% agarose gel for 60 min at 160 V. Visualization was done using the UVIPro Gold transilluminator (UVItec, UK).

### G6PD Genotyping

G6PD genetic variants that confer resistance to severe malaria including 202, 376, 542, 680, and 968 A-deficiency polymorphisms referred as G6PD202, G6PD376, G6PD542, were evaluated after performing HPLC and HbS molecular Analysis in 100 randomly selected DNA samples belonging to the five more frequent ethnic groups (Balanta, Fula, Papel, Manjaco, Mandingo), and in HbS gene carriers who had DNA remaining for the analysis.

The coding sequence of exons analyzed of the G6PD gene, Xq28 (NM_X03674; OMIM#305900), for G202A, A376G, A542T, T968C polymorphisms, was PCR-amplified with 30-cycle reactions at 94°C for 1 min, at 58°C for 1 min and at 72°C for 1 min. The reaction was done in a final volume of 50 μl comprising 50 ng DNA, Buffer II 10X, MgCl_2_ Solution 25 mM, dNTP's 10 mM, Primers 100 μM, AmpliTaq Gold DNA Polymerase 5 u/μl.

The amplification products for G6PD202, G6PD376, G6PD542, G6PD968 polymorphisms were subjected to direct sequencing (3500Dx Genetic Analyzer, Applied Biosystems), instead the product PCR for G6PD680 was digested with BstNI restriction enzyme (New England, BioLabs): 4 μl PCR product with 2 μl of enzyme at 60°C for 15 min.

## Results

Eight doctors and three technicians from the two hospitals were trained on SCD and sampling techniques through several on site meetings. Accessing the two hospitals consecutively, 848 children (503 from BCH 345 from HRF) were tested comprising of 498 males and 350 females, with a mean age of 6.8 years (range: 1–16). The children belonged to the most frequent ethnic groups of Guinea Bissau (Table 1). The families all resided in the capital city and came from different parts of town.

**Table 1:** Basic demographics of the pediatric population in the two hospitals.

**Variables**	**Total**		**BOR**		**HRF**	
	**N**°****	**%**	**N**°****	**%**	**N**°****	**%**
	848	100	503	59,3	345	40,7
**Gender**
Male	498	58.7	294	58.4	209	60.6
Female	350	41.3	204	40.5	141	40.9
**Age (years)**
Median	6.8		6		8	
**Ethnic groups**
Balanta	136	16	82	16.3	54	15.6
Fula	121	14.27	73	14.5	50	14.5
Papel	123	14.50	82	16.3	41	11.9
Mandingo	111	13.08	41	8.1	70	20.3
Manjaco	75	8.84	49	9.7	26	7.5
Mancanha	66	7.78	39	7.7	40	11.6
Biafada	50	6.01	4	0.7	46	13.3

*BCH, Clinica Pediátrica Bor; HRF, Hospital Raoul Follereau*.

The blood samples collected on absorbent paper were of excellent quality.

### HPLC and Molecular Analysis

HBB molecular analysis identified 8 individuals with Hb SS SCD (0.94%) (mean age 7.27 years), 59 individuals with HbSA (6.95 %) (mean age 6.05 years), 2 individuals with HbAC (0.23%) (mean age 2 years), 1 individual bearer of a sequence variant samesense (0.19 %) (8 years). None had HbSC or HbSβthalassemia, frequent in other West African countries.

HbS allelic frequency was 4.42%. Details are given in Table 2.

**Table 2:** Genotype and hemoglobin F %.

**Genotypes**	**Samples (***n***)**	**Patient's age (Median)**	**Hemoglobin F (%)**	**Hemoglobin range (g/dl)**
AS	59	6.05	8.26	7.6–16.2
SS	8	7.27	9.15	8–10.3
AC	2	2	8.95	5.1–12.8

A significant difference in the frequency of HbS was found between ethnic groups: Fulas 25.42%, Mandingas 18.64%, Pepel 11.86%, Mandjau 6.77%, Biafada 13.55%, 10.16% Balanta, and 11.86% the others (Table 3).

**Table 3:** Distribution of abnormal hemoglobin in the different ethnic groups.

	**HbAS**		**HbSS**		**HbAC**	
**Variables**	**N°**	**%**	**N°**	**%**	**N°**	**%**
Total	59	6.95	8	0.94	2	
Median Age (*years*)	6.05		7.27		2	
**Ethnic group-*****n*** **(%)**
Balanta	6	10.16	1	12.5		
Fula	15	25.42	1	12.5	2	100
Papel	7	11.86	1	12.5		
Mandingo	11	18.64	3	37.5		
Manjaco	4	6.77	1	12.5		
Mancanha	1	1.69				
Biafada	8	13.55				
Others	7	11.86	1	12.5		

### Hemoglobin Haplotypes

Thirty-two patients with the HbS gene had enough genetic material to be analyzed for the SCD haplotype background: 5 HbSS and 27 HbAS. Sixty-four chromosomes were therefore analyzed.

The Senegal haplotype was the most prevalent of all 5 SCD haplotypes with 31% (*n* = 20) followed by Cameroon haplotype with 11% (*n* = 7), with 1 chromosome from the Benin haplotype (2%). There was neither the Indian-Arab nor Central African haplotypes in this study (Supplementary Table 1; Supplementary Figure 2). The proposition of chromosomes with the atypical haplotype was surprisingly high (56%; *n* = 30) with 83.3% (*n* = 30) positive for the HinfI(5'β) restriction site.

### G6PD Molecular Analysis

One hundred forty-one samples were analyzed for G6PD: 100 randomly selected samples of the five more frequent ethnic groups (20 each from Balanta, Fula Pepel, Mandjaco, Mandingo) with HbAA genotype, 37 samples from individuals with HbAS, and 4 samples from HbSS individuals (details in Supplementary Table 2) according to the availability of DNA.

Mutational analysis with PCR and direct sequencing of the G6PD gene showed the presence of 376G and 968G alleles, not 202A, 542T, and 680T.

376G allele is 35% of 100 samples belonging to five ethnic groups more frequent in the population, and 21.6% of HbAS carriers with allelic frequency, respectively, of 24 and 14.8%.

G6PD molecular analysis in HbSS samples gave no significant results.

Interestingly, differences are noted between different ethnic groups: 11/35 (31.4%) 376G individuals are Fulas (1 homozygous female, and 5 heterozygotes, 5 hemizigous males); 8/37 (21.6%) HbAS samples have 376G and 3/8 (37.5%) are Fulas.

Allele 968G is 8% of 100 samples evenly distributed between five ethnic groups (1 homozygous female, and 3 heterozygotes, 4 hemizigous males), 2.7% of SCD HbAS and 25% of HbSS patients, 37.5% of AS and 25% of SS are Fulas. The percentage of 376G/968G is the same between different ethnic groups.

**Figure 1:**
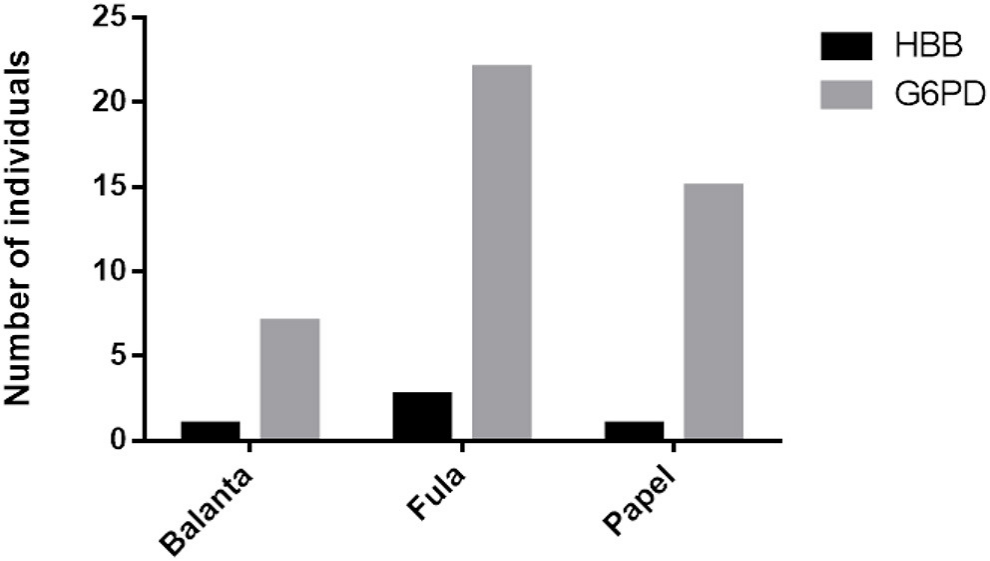
Distribution of the HbS allele (HBB mutation) and the G6PD mutation in the three main ethnic groups: Balanta, Fula, and Papel.

Figure 1 displays the distribution of G6PD mutation and HbS allele in the three main ethnic groups.

## Discussion

In this first study after the publication of the 2008 UN recommendation to strengthen SCD programs throughout the world (21), we found higher prevalence of HbS allele in a pediatric population in Bissau, Guinea-Bissau, compared to previous studies (15), confirming the need to develop nationwide SCD screening and comprehensive care programs in the country. Moreover, our results demonstrate the feasibility of good sample collection by staff that has been adequately trained within the framework of North-South and South-South international collaborative projects.

The overall HbS allelic frequency was 4.42%, lower than neighboring countries. The prevalence of HbSA carriers also seems lower, which was 6.95% (12–14). Interestingly, the prevalence of the carrier state was different in the various ethnic groups, being higher in the Fula and the Mandingo. Minor differences were also observed among ethnic groups in the neighboring Republic of Guinea (22), although not as high. Specific characteristics of Guinea Bissau might justify this data. It is a very secluded country, with less connections to the other West African countries and to Europe, with a widely spread rural population and significant numbers of interfamily marriages between some of the ethnic groups. The country is therefore not new to unique epidemiological characteristics. It was, for example, the only country in which HIV 2 was more widespread than HIV 1 in the past decades (23); and multidrug resistant uberculosis (TB) has lower incidence than in other West-African countries (24). Furthermore, the very high maternal and infant mortality rates of Guinea Bissau (child mortality rates are the highest worldwide at 152.5 per 1,000 livebirths) (25), as well as the increased risk of stillbirth in SA carriers (26), could justify a higher number of children with SS dying earlier and not being diagnosed. Also, there is a lower rate of HbSA children being born from S carrier mothers. Moreover, the relatively high mean age of our pediatric cohort (mean age 6.8 years) and of the children with SCD (mean age 7.27 years) could have excluded children with SCD who might have died before 5 years of age (4). Taken together, these data reinforce the need for screening early in life, such as in newborn screening programs, in Guinea Bissau.

It is also often difficult to know exactly how the coupling of individuals in a population happens, in particular, it is difficult to know if a population presents the rule of panmixy. If there is genetic isolation between subpopulations, it is possible that there is also a certain differentiation of allele frequencies, so the population shows a deviation from the Hardy-Weinberg equilibrium, even if the balance is respected within each population (27).

While the different ethnic distribution of the HbS allele warrants a deeper understanding of the genetic background of the population and further research (28–30), it can also aid to prioritize actions for SCD, such as starting awareness campaigns and screening in areas of the country or sectors of the city where the highest rate of carriers is living. Unfortunately, Guinea Bissau has very limited resources allocated to health care and other urgent health priorities like infectious diseases -HIV, TB, and cholera which catalyzed health care energy and resources in the past years. No resources were available for non-communicable diseases. Implementing screening in a stepwise manner with a priority list could be beneficial in this limited resource setting.

The co-existence of the HbS allele and the G6PD mutation in the setting of Guinea Bissau is important for phenotypic variability of hematological diseases. In fact, G6PD has been shown to influence severity of hemolysis and cerebrovascular manifestations (31–33). Moreover, the G6PD 376G allele is present in 35% of the HbAA samples and in 21.6% of the HbAS carriers, with an allelic frequency of 24 and 14.8%, respectively. The prevalence of G6PD genotypes in HbSS and HbSA did not differ (*p* > 0.05) from those found in the controls. The prevalence of G6PD deficiency did not change when patients were stratified by age, suggesting that there is no advantage of the association of G6PD deficiency with HbSS.

The Senegal haplotype is the most prevalent in our cohort, followed by the Cameroon haplotype. This could justify the relatively high percentage of HbF found in our cohort as it is well known that the Senegalese haplotype presents higher HbF (34). However, there is also a large proportion of atypical haplotypes in this cohort, which may be indicative of a genetically heterogeneous population that may have high levels of admixture or be a site of early migration. In fact, atypical haplotypes not belonging to the 5 most common ones (Senegalese, Benin, Bantu, Cameroon, Arab), have been described in other populations (Brazil, Nigeria, India) and could be responsible for the extreme phenotypic diversity of SCD (34–36). Future genetic studies are needed in this field.

Our study has several limitations. First of all, the sample collection involved only children living in the capital city of Bissau and occurred in a timeframe of only 8 months due to limited funding and staff capacity. Therefore, a wider sample, including also children from rural areas might be useful to estimate the real nationwide prevalence. Secondly, the organization of the blood sample analysis was complex and cannot be reproduced on large scale. Today, several rapid tests have become available and allow point-of care testing through a less complex diagnostic pathway than the one evaluated in this study. Nevertheless, the prevalence of HbS carriers and HbSS was high and warrants the development of a newborn screening program in Guinea Bissau. The possibility to screen with the different types of rapid tests that are now on the market (37) will hopefully enhance the screening capacity, both in the urban and rural areas of Guinea Bissau, as a pilot project has demonstrated (38).

## Conclusion

This was the first study where data on sickle cell trait and G6PD deficiency frequencies were obtained for Guinea-Bissau pediatric populations. These data contribute to the evidence of the need to develop a SCD screening program in the country. Moreover, our results support the need to further explore the genetic background of various SCD populations across Africa in order to contextualize the heterogeneous clinical phenotypes seen across the continent. Such diverse populations are ideal for more precise haplotyping techniques to investigate the genomic background of SCD in West Africa, where there is an apparent lack of literature on SCD and the understanding of genotype-phenotype correlations.

## Data Availability Statement

The raw data supporting the conclusions of this article will be made available by the authors, without undue reservation.

## Ethics Statement

The studies involving human participants were reviewed and approved by Ethics Committee of the Hospital RAoul Follereau in Guinea Bissau; Ethics Committee of the Province of Padova, Padova, Italy. Written informed consent for participation was not required for this study in accordance with the national legislation and the institutional requirements.

## Author Contributions

RC, FR, and MM designed the study. DC, AA, and SD collected the samples. MC, MM, FM, AW, GP, and GV performed the analysis. RC wrote the manuscript. All authors reviewed the manuscript. All authors contributed to the article and approved the submitted version.

## Conflict of Interest

The authors declare that the research was conducted in the absence of any commercial or financial relationships that could be construed as a potential conflict of interest.

## Publisher's Note

All claims expressed in this article are solely those of the authors and do not necessarily represent those of their affiliated organizations, or those of the publisher, the editors and the reviewers. Any product that may be evaluated in this article, or claim that may be made by its manufacturer, is not guaranteed or endorsed by the publisher.
