# Supplementary material for: Distribution of HbS Allele and Haplotypes in a Multi-Ethnic Population of Guinea Bissau, West Africa: Implications for Public Health Screening
Source: Front Pediatr. 2022 Apr 7;10:826262. doi: 10.3389/fped.2022.826262 (PMC9021572; doi:10.3389/fped.2022.826262)
Supplement: Supplementary file 1 [file Data_Sheet_1.docx]

**Supplementary Material**

Supplementary Figure 1. Map of Guinea Bissau


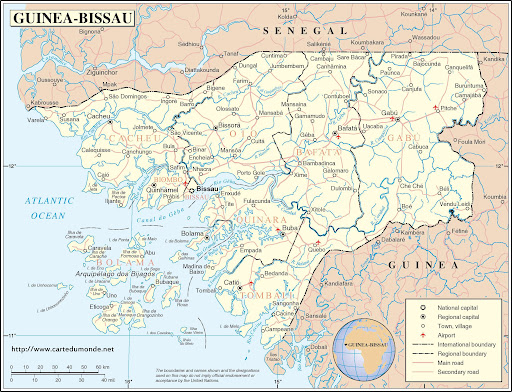


Supplementary Table 1. Sickle Cell Disease haplotype frequencies in patients from Guinea Bissau

| **Haplotype** | **Chromosomes (n)** | **Percentage**  **(%)** |
| --- | --- | --- |
| Senegal | 20 | 31,25 |
| Cameroon | 7 | 10,93 |
| Benin | 1 | 1,56 |
| Atypical | 36 | 56,25 |
| TOTAL | 64 | 100 |

Supplementary Table. 2 Samples on which G6PD analysis was performed and ethnic origin.

| Genotypes | Ethnic Group | | | | | | | | |
| --- | --- | --- | --- | --- | --- | --- | --- | --- | --- |
|  | Balanta | Fula | Papel | Manjaco | Mandingo | Biafada | Susso | Others | Total (n) |
| AA Mixed Random | 20 | 20 | 20 | 20 | 20 |  |  |  | 100 |
| AS | 4 | 13 | 4 | 2 | 4 | 6 | 1 | 3 | 37 |
| SS | 1 | 1 | 1 |  | 1 |  |  |  | 4 |

Supplementary Figure 2. Distribution of Haplotypes
